# Quantitative Risk Assessment of Exposure to *Mycobacterium avium* subsp. *paratuberculosis* (MAP) via Different Types of Milk for the Slovenian Consumer

**DOI:** 10.3390/foods11101472

**Published:** 2022-05-18

**Authors:** Tanja Knific, Matjaž Ocepek, Andrej Kirbiš, Branko Krt, Jasna Prezelj, Jörn M. Gethmann

**Affiliations:** 1 Institute of Food Safety, Feed and Environment, Veterinary Faculty, University of Ljubljana, Gerbičeva ulica 60, 1000 Ljubljana, Slovenia; andrej.kirbis@vf.uni-lj.si; 2 Institute of Microbiology and Parasitology, Veterinary Faculty, University of Ljubljana, Gerbičeva ulica 60, 1000 Ljubljana, Slovenia; matjaz.ocepek@vf.uni-lj.si (M.O.); brane.krt@vf.uni-lj.si (B.K.); 3 Department of Mathematics, Faculty of Mathematics and Physics, University of Ljubljana, Jadranska ulica 19, 1000 Ljubljana, Slovenia; jasna.prezelj@fmf.uni-lj.si; 4 Department of Mathematics, Faculty of Mathematics, Natural Sciences and Information Technologies, University of Primorska, Glagoljaška 8, 6000 Koper, Slovenia; 5 Institute of Mathematics, Physics and Mechanics, Jadranska ulica 19, 1000 Ljubljana, Slovenia; 6 Friedrich-Loeffler-Institut, Federal Research Institute for Animal Health, Institute of Epidemiology, Südufer 10, 17493 Greifswald-Insel Riems, Germany; joern.gethmann@fli.de

**Keywords:** *Mycobacterium avium* subsp. *paratuberculosis* (MAP), quantitative risk assessment, Monte Carlo simulations, food safety, milk

## Abstract

This study aimed to assess the risk of exposure to *Mycobacterium avium* subsp. *paratuberculosis* (MAP) via milk for the Slovenian consumer. MAP is suspected to be associated with several diseases in humans, therefore the risk of exposure should be better understood. The primary source of MAP for humans is thought to be cattle, in which MAP causes paratuberculosis or Johne’s disease. We developed a stochastic quantitative risk assessment model using Monte Carlo simulations. Considering the assumptions and uncertainties, we estimated the overall risk of exposure to MAP via milk to be low. For people consuming raw milk from MAP positive farms, the risk was high. On-farm pasteurisation reduced the risk considerably, but not completely. The risk of exposure via pasteurised retail milk was most likely insignificant. However, with a higher paratuberculosis prevalence the risk would also increase. Given the popularity of raw milk vending machines and homemade dairy products, this risk should not be ignored. To reduce the risk, consumers should heat raw milk before consumption. To prevent a potential public health scare and safeguard farmers’ livelihoods, a reduction in paratuberculosis prevalence should be sought. Our results show that culling clinically infected cows was insufficient to reduce milk contamination with MAP.

## 1. Introduction

*Mycobacterium avium* subsp. *paratuberculosis* (MAP) is assumed to be associated with Crohn’s disease (CD), a chronic inflammatory bowel disease [1] and several other chronic diseases in humans, for example type 1 diabetes mellitus [2,3], Hashimoto thyroiditis [4], multiple sclerosis [5], Parkinson’s disease [6], and Alzheimer’s disease [7]. In addition, immunocompromised individuals such as HIV patients [8] and children [9] have been recognised as vulnerable populations [10]. MAP is an obligate intracellular multi-host pathogen, with the main reservoir and host likely to be ruminants [11,12]. In cattle, MAP causes paratuberculosis or Johne’s disease [13], a globally endemic disease with major economic consequences [14]. The disease has a slow course with a long incubation period and usually occurs as a subclinical infection [11]. Clinically, it manifests as chronic and progressive gastroenteritis [15]. Calves are most susceptible, yet infections in adult animals occur more frequently than previously thought [16]. Whittington et al. [17] reported that about half of the 48 countries that participated in their survey had a herd-level prevalence of more than 20% and in some countries the prevalence was more than 40%. The authors pointed out that underestimation and under-reporting are common [17].

The main reasons for the association between MAP and CD are: (1) the clinical signs and tissues affected are very similar to paratuberculosis in cattle [13], (2) MAP can infect many domestic and wild animal species, including non-human primates [18], (3) MAP has been isolated from patients with CD and significant associations have been demonstrated [19,20], and (4) successful antibacterial drug treatments [21,22]. However, the lack of correlation between CD and isolation of MAP has also been reported [23]. Some researchers hypothesise that MAP may not be the cause of human diseases, but rather an opportunistic pathogen [12] or a possible trigger of disease [5]. Despite the growing number of studies linking MAP to human diseases, the debate on whether or not MAP is a zoonosis remains contentious [24,25,26]. In contrast, many researchers believe that there are reasons to suspect that it poses a risk to human health [27,28].

There is limited data on the prevalence and incidence of CD in Slovenia, but it is estimated that there were about 2000–2500 patients in 2016 [29]. The study on the incidence rate of paediatric inflammatory bowel disease revealed that the annual incidence of CD per 100,000 children and adolescents in Slovenia increased from 3.6 in 2002 to 4.6 in 2010 [30]. While CD incidence has stabilised in countries with traditionally high prevalence, overall prevalence continues to rise worldwide. This represents a significant economic burden, with annual direct health care costs and loss of labour productivity estimated at around 5400 € per patient [31]. Even though details about the nature and consequences of MAP infection are not known, it is important to understand the risks of human exposure better.

Probably the most important source of human exposure to MAP is infected cattle and their food products [32]. Many potential sources of human exposure have been identified, namely contaminated food (e.g., milk and dairy products, meat), water, environment, and direct contact with infected animals [33]. Waddell et al. [33] noted that people eating a normal diet could have repeated exposure to MAP. Although potential exposure has been identified, there are still many uncertainties and unknowns associated with MAP as a risk to human health. For example, the relative importance of each route of exposure, the minimum infectious dose, and the duration of exposure are unknown. There is also limited knowledge about infectious doses for animals. Experimental and natural exposure studies have investigated different doses, the number of doses, the route of infection, different endpoints, and animal species of different ages and breeds. However, there is still no clear infectious dose and no data to assess the consequences of long-term exposure to low doses. The most studied is the exposure outcome in cattle, however the only conclusion on which most studies agree is that infection dynamics are very complex and appear to depend on multiple pathogen and host-related factors that vary between and within hosts. Investigation of exposure outcomes is further complicated by the long incubation period, poor diagnostics, and intermittent shedding [34,35]. In cattle, doses as low as 10^3^ MAP cells have been associated with infections [36]. In experimental studies, doses ranging from 1.5 × 10^6^ to 3 × 10^10^ MAP cells have been found to cause infection when administered once or several times [34,37]. In sheep, even lower doses have caused infection [37]. Considering that already latently and subclinically infected animals can shed MAP in varying amounts and that clinically infected animals can excrete up to 10^9^ CFU per gramme of faeces [38], these infection doses do not seem high. Factors associated with infection and its severity appear to be species, breed, infectious dose and number of doses, age, route of infection as well as MAP strain [37]. The relative importance of the different routes of transmission, the minimum infectious dose for animals of any age, and shedding pattern of infected animals are still gaps that hinder the prevention and control of paratuberculosis [39]. The variations in exposure outcomes and complex disease dynamics described for paratuberculosis in cattle are also found in the human mycobacterial diseases tuberculosis and leprosy. All three diseases have been found to share some candidate immune genes that overlap with those found in CD [35].

The most-studied sources of human exposure are milk and dairy products. Milk can be contaminated directly by cows excreting MAP in the milk or indirectly by faecal contamination and dirt from the udder, milking machines, or other surfaces. Even when good hygiene standards are maintained, this is possible [40]. However, a study by Gerrard et al. [41], which examined the presence of viable MAP and its levels in bulk milk samples, suggested that the main source of milk contamination is direct shedding in the udder. MAP was cultured from raw milk, pasteurised milk [41,42], retail cheese made from pasteurised and raw milk [33], and powdered infant formula [43]. The extent of contamination of various milk and dairy products varies widely between studies. In a study conducted in England in 2014 to 2015, semi-skimmed pasteurised retail milk was sampled and viable MAP cells were found in 10.3% of samples [41]. On the other hand, there are also studies in which MAP was not found in the above mentioned products [44]. Nevertheless, in both cases, the problem with diagnostic tests should not be ignored. It is unknown what proportion of human exposure to MAP milk and dairy products accounts for [45] but some countries are already implementing measures to reduce the risk of human exposure [17].

It has been demonstrated that MAP can survive many food processing steps, including the pasteurisation of milk [41,42]. When pasteurised with the holder method (at 63 °C for 30 min), the inactivation of MAP by heat treatment time is not linear and shows tailing due to the presence of MAP cells in clumps, which are more heat resistant than single cells [46,47]. Gerrard et al. [41] hypothesised that the clustered MAP cells may primarily reside within the somatic cells where they are better protected during heat treatment, resulting in low levels of MAP in retail milk. It has also been observed that prolonged exposure to heat is more effective than higher temperatures. Thus, some MAP cells may remain intact after high-temperature short-time pasteurisation if present in sufficiently high concentrations in the milk [46,47]. Fechner et al. [48] conducted an experimental study investigating on-farm commercial high-temperature short-time pasteurisation (at 73.5 °C for 20 to 25 s) of spiked fresh raw milk intended for calf feeding. The initial concentration of MAP in the milk was 10^7^ and 10^4^ MAP cells/mL, which was significantly reduced by pasteurisation. Nevertheless, it was found that about 10^3^ MAP cells/mL remained viable. However, ultra-high temperature (UHT) milk treatment is thought to deactivate MAP completely [47].

Garcia et al. [49] pointed out that if a link between MAP and CD is confirmed in humans, significant economic losses in the livestock sector can be expected due to public health scares. The losses would disproportionately affect the dairy sector, making paratuberculosis control programmes economically beneficial and increasing farmers’ willingness to participate [49]. This is particularly important for the dairy sector in Slovenia, as milk production accounts for almost one-third of total livestock production [50].

The objective of this study was to perform a quantitative risk assessment to evaluate the risk of exposure to MAP in different types of milk for Slovenian consumers. We hypothesised that the risk of exposure is not negligible, as we believed that the prevalence of MAP in Slovenia is higher than assumed in previous estimates. In 2008, an estimated 18.49% of cattle herds were infected, while the true prevalence at the animal level was estimated at 3.96% [51]. We used modelling to overcome the lack of empirical data and the problem of inadequate testing to diagnose MAP infections, as it allows the use of published information and expert opinion.

## 2. Materials and Methods

To estimate the potential human exposure to MAP via different types of milk in Slovenia, we performed a stochastic quantitative risk assessment using Monte Carlo simulations. We focused on different milk types that were of greater importance due to production and consumption trends in Slovenia, namely raw milk, pasteurised milk, and UHT milk. Nevertheless, we were limited by the lack of data on MAP persistence during milk processing. We did not consider the import or export of raw milk and milk ready for consumption, as studying the dairy industry of importing countries was beyond the scope of this study.

Modelling allowed the use of available data on Slovenian cattle population, movements, and production, as well as published data on MAP epidemiological characteristics. Where necessary or deemed appropriate, the data were supplemented with expert assessments.

### 2.1. Model Structure and Parameters

In this risk assessment model, we used (1) the results of a compartmental model of MAP spread in a typical Slovenian dairy herd [52], (2) the results of a temporal network model of MAP spread between herds in Slovenia [53], (3) available data on the situation of dairy farming in Slovenia and (4) data from the literature. The risk assessment was performed in Microsoft Excel 365 using the add on @RISK, version 8.0 [54]. We obtained and analysed data from the Statistical Office of the Republic of Slovenia (SURS) [55], the Agricultural Institute of Slovenia [56], the Agency of the Republic of Slovenia for Agricultural Markets and Rural Development and the Chamber of Commerce and Industry of Slovenia. All data used refer to the year 2018. We fitted distributions to the data on the herd size, milk yield per cow, herd level prevalence, and proportion of subclinically and clinically infected cows. We selected the distributions with the lowest Akaike Information Criterion (AIC) value. There are several statistics for choosing the best-fitted distribution for the given data. We chose the AIC criterion because it considers the log-likelihood function and the number of estimated parameters for the selected distribution [57]. The distributions for other parameters were taken from the literature and some were supplemented or estimated based on expert opinion. All probability distributions with their values for the input variables are listed in Table 1.

**Figure 1:**
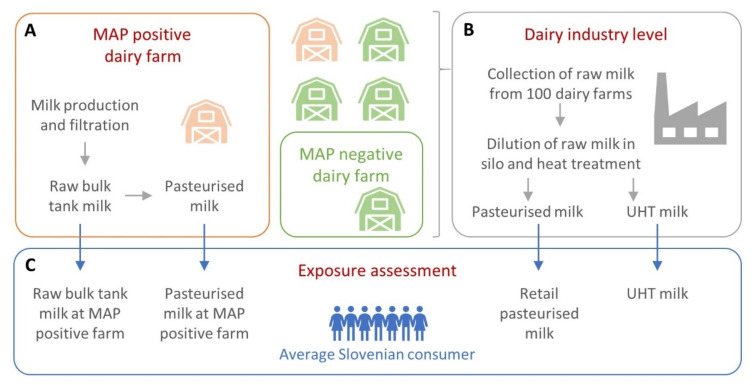
Schematic structure of the model for quantitative risk assessment of exposure to *Mycobacterium avium* subsp. *paratuberculosis* (MAP) via different types of milk for the Slovenian consumer. The model consists of three parts: (**A**) a farm-level sub-model, (**B**) a dairy industry-level sub-model, and (**C**) a potential human exposure sub-model.

Our human exposure risk assessment model consists of three parts: a farm-level sub-model, a dairy industry-level sub-model, and a potential human exposure sub-model. The schematic structure of the model and the milk types considered are shown in Figure 1.

The farm-level sub-model was used to estimate the extent of contamination of bulk tank milk in a MAP positive farm. The number of cows per herd was simulated using a Pearson distribution of type VI with an average of 17 cows per herd (*N*). As mentioned above, this distribution was fitted based on the real data using the AIC value. Then the number of healthy, subclinical, and clinical cows was calculated for this herd size. Since the dynamics of the spread of MAP within the herd differ between small and large herds, we included two options for calculating the proportion of clinical and subclinical cows (*P_s_*, *P_c_*) based on expert opinion. The selected threshold for a small herd was 50 cows. Consequently, if the model simulated fewer than 50 cows per herd, we used the results of our compartmental model. However, if 50 or more animals were simulated, we assumed a distribution based on published data and expert opinion.

The milk produced per healthy cow (*m_h_*) was simulated using a lognormal distribution with an average of 20.59 litres of milk per cow per day. The reduction in milk production per subclinical (*m_s_*) and clinical cow (*m_c_*) was assumed and estimated based on published literature and expert opinion. Triangular distributions for excretion of MAP in milk from subclinical (*MAP_ms_*) and clinical cows (*MAP_mc_*) were determined by the expert based on published estimates. The distributions for milk contamination with MAP due to faecal contamination by subclinical (*MAP_fs_*) and clinical cows (*MAP_fc_*) were taken from the literature. Internal milk contamination (*MAP_int_*) and external milk contamination (*MAP_ext_*) were then calculated using herd-level (*m*) milk production. The final MAP contamination of raw bulk tank milk (*MAP_herd_*) was reduced by up to 67% (*φ*) by filtering the milk. The calculations are shown in Table 2.

The second part was the dairy industry-level sub-model in which we modelled the dilution of MAP in the milk silo. Because we did not have information on which farms supplied milk to the same dairy plant, we assumed a random selection of farms and therefore did not care about the size of the silo. However, we calculated the number of farms for the simulation based on a silo with a capacity of 30,000 L. We also assumed that farms supplying milk to the dairy had at least three dairy cows. We simulated milk collection in a silo using one hundred farm level sub-models (*MAP_industry_*). To determine each simulated herd’s infection status (*HS*), we used fitted distributions of herd prevalences from the temporal network model (*S_i_*). However, we neglected the worst-case scenario, as it was considered highly unlikely that more than 50% of the herds in Slovenia would be MAP positive. The model assumed a random distribution of positive herds.

The third part combines the results from the first two parts with data on MAP reduction during processing and consumption to model the MAP contamination of milk at the farm and dairy industry level and thus the potential human exposure. We simulated MAP contamination of raw milk and pasteurised whole milk for positive farms and at the dairy industry level for the three herd-level prevalence scenarios. The pasteurisation process reduced MAP by at least 4 to 7 log colony forming units (CFU) (*ε*). The terms MAP CFU and MAP cell are used interchangeably. We used the term MAP CFU to refer to the unit of detection of viable MAP, while the term MAP cell refers to the actual number of individual MAP. The random process of distributing MAP cells in bulk tank milk (*MAP_herd raw_*, *MAP_herd past_*) or silo (*MAP_industry past_*) was considered using Poisson distribution.

The results are expressed as MAP contamination of different types of milk with the distribution of MAP cells per litre, proportions of MAP positive litres of milk or litres of milk with >0 MAP cells, and proportions of litres of milk with >100 MAP cells per litre. These values were chosen based on previous studies [62] and did not reflect the risk that MAP could pose to human health, as the infectious dose to humans (assuming that MAP is a zoonosis) is not known.

Finally, we calculated the annual exposure of an average Slovenian consumer who consumed 43 litres of milk in 2018. Since consumption is reported for all milk types combined, we calculated the average annual consumption of the different milk types based on the shares on the market, assuming that the average consumer consumes all these milk types. According to SURS data for 2018, on-farm consumption and direct sales of raw milk amounted to more than 13 million litres of milk [55]. In the same year, the Chamber of Commerce and Industry of Slovenia recorded that dairies produced more than 16 million litres of pasteurised milk and almost 150 million litres of ultra-high temperature treated (UHT) milk.

### 2.2. Model Simulation and Validation

We ran 10,000 iterations of Monte Carlo simulations with Latin hypercube sampling using the programme @RISK, version 8.0 [54]. Monte Carlo simulations are repeated computations of our model, with each iteration based on a random sample of defined probability distributions of the input variables. We chose Latin hypercube sampling, which forces sampling from all parts of the distribution. This is stratified sampling, which means that each probability distribution of the input variables is divided into equal parts or strata. A random sample is then drawn from each stratum, whereas in traditional Monte Carlo sampling, a random sample is drawn directly from probability distributions. Therefore, to achieve the same level of accuracy, Latin hypercube sampling requires fewer iterations of simulations than the Monte Carlo sampling method [57].

The validation consisted of two parts. The first part was internal validation. The internal validation included a thorough review of all underlying assumptions, calculations, and parameters. Based on an analysis of Slovenian census data from various sources and the literature on the epidemiological characteristics of MAP, T.K. proposed the initial model assumptions and parameters. As the formal review of the literature was beyond the scope of this study, not all reviewed studies are presented. Only the studies that were used to determine the parameters or that most strongly supported the expert opinion are presented. Expert opinions are consolidated opinions of several experts. During the development and analysis of the model, J.M.G., a recognised specialist in epidemiology, repeatedly reviewed the assumptions, calculations, and parameters. The assumptions and parameters were reviewed by two Slovenian experts on paratuberculosis, M.O. and B.K. Additionally, the calculations and assumptions were checked by J.P., a mathematician. Finally, the assumptions, results, and their interpretation were discussed with all co-authors and compared with other studies.

The second part of validation was sensitivity analysis. The inputs with the highest impact on the model’s outputs were identified through sensitivity analysis. The sensitivity analysis results show the sensitivity of the output variables to the distribution of each input in the model. In addition, we tested the impact of different threshold values for the small herd size.

### 2.3. Risk Characterisation

The final step in our risk assessment is risk characterisation. The exposure assessment results are summarised and presented in qualitative terms based on the terminology proposed by WHO/FAO [63] and EFSA [64]. For the qualitative expression of the likelihood of human exposure to MAP, we used the terms: insignificant—not expected to occur, rare—may occur only in exceptional circumstances, unlikely—could happen at some time, possible—could occur or should occur at some time, likely—likely to occur in most circumstances, and almost certain—expected to occur in most circumstances. We defined effects or consequences based on the average concentration of MAP per litre of milk, using the terms: insignificant (0 MAP CFU/L), low (1–10 MAP CFU/L), moderate (11–100 MAP CFU/L), and high (>100 MAP CFU/L). The risk level was negligible, low, moderate, high, and very high, depending on the determined probability of human exposure and the average MAP concentration per litre of milk. We indicated the risk level for each evaluated milk type separately and the overall risk for the average Slovenian consumer.

In addition, we expressed the level of uncertainty using the qualitative terms low, moderate, or high, meaning that there is sufficient, some, or little scientific evidence to support the assumptions and results of our models. In discussion, we identified the assumptions and uncertainties used and described their impact on the results and the interpretation of our findings.

## 3. Results

### 3.1. MAP Contamination of Raw and Pasteurised Milk in a MAP Positive Farm

**Figure 2:**
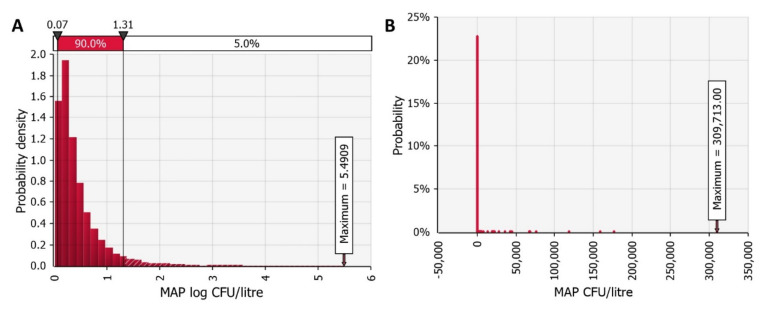
*Mycobacterium avium* subsp. *paratuberculosis* (MAP) contamination of raw milk in a MAP positive farm. (**A**) Probability density of MAP contamination of raw bulk tank milk. (**B**) Discrete probability of MAP contamination per litre of raw milk.

MAP concentration in a positive farm (*MAP_herd_*) ranged from 0.01 to 5.49 log CFU per litre of raw bulk tank milk. The mean concentration per bulk tank milk was 0.46 log CFU per litre with a standard deviation (SD) of 0.48 log CFU per litre. MAP concentration in a litre of raw milk (*MAP_herd raw_*) varied from zero to a maximum of nearly 310 thousand MAP CFU per litre. On average, one litre of raw milk in a positive farm contained 164 MAP CFU with a 90% confidence interval (CI) of ±77.58 MAP CFU and 5th and 95th percentile (PC) of zero and 22 MAP CFU per litre, respectively. The mode was one, and the median was two MAP CFU per litre of raw milk. The model estimated that 86.67% of litres of milk contained MAP cells, of which 1.83% contained at least 100 MAP cells. The probability distributions of raw milk contamination with MAP are shown in Figure 2. 

**Figure 3:**
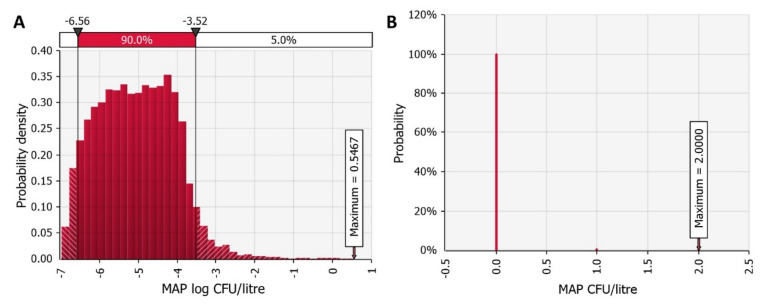
*Mycobacterium avium* subsp. *paratuberculosis* (MAP) contamination of pasteurised milk in a MAP positive farm. (**A**) Probability density of MAP contamination of pasteurised milk at the farm level. (**B**) Discrete probability of MAP contamination per litre of pasteurised milk.

After on-farm pasteurisation (*MAP_herd past_*), the estimated proportion of litres of milk containing MAP cells was much lower; only 0.08% litres of milk contained MAP cells. Figure 3 shows the probability distributions for contamination with MAP in pasteurised milk at the farm level. The mean MAP concentration in pasteurised milk at the farm level was −5.33 log CFU per litre with a 90% CI of ±0.02 log CFU per litre and ranged from −6.95 to 0.55 log CFU per litre. The estimated maximum contamination of a litre of pasteurised milk in a MAP positive farm was two MAP cells. The mean contamination was 0.001 and the 99th PC was zero MAP cells per litre of milk.

### 3.2. MAP Contamination of Pasteurised and UHT Milk at the Industry Level

**Figure 4:**
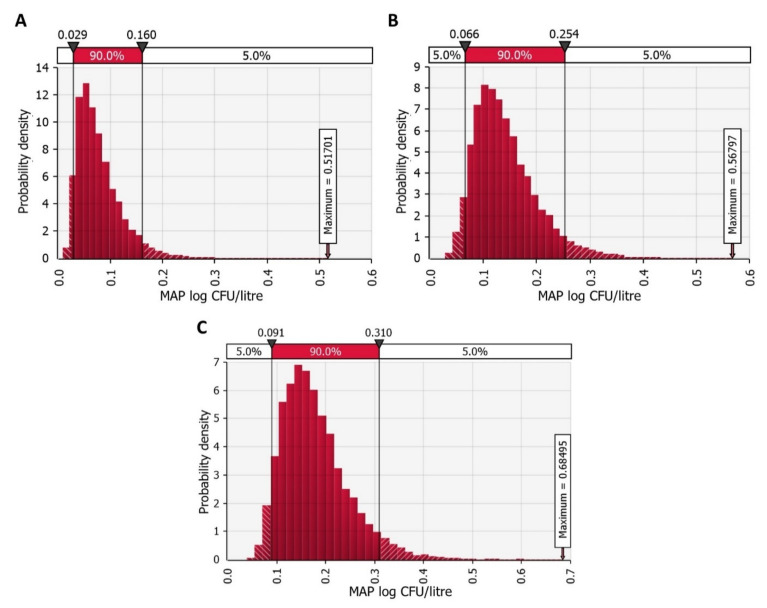
*Mycobacterium avium* subsp. *paratuberculosis* (MAP) contamination of pasteurised milk at the dairy industry level using three scenarios of mean paratuberculosis prevalence: (**A**) scenario 1 with 21.3% prevalence, (**B**) scenario 2 with 38.89% prevalence, and (**C**) scenario 3 with 49.21% prevalence.

We simulated raw milk collection from one hundred dairy farms with three herd level paratuberculosis prevalence scenarios at the industry level. These were scenarios 1, 2 and 3 with an average paratuberculosis prevalence of 21.3%, 38.89%, and 49.21%, respectively, as estimated by the temporal network model of MAP between herd spread in Slovenia [53]. When the herd was determined to be MAP negative in the present model, the model simulated the amount of milk produced free of MAP. Therefore, we simulated the reduction of MAP contamination due to the dilution of MAP in the milk silo (*MAP_industry_*). Figure 4 shows the probability density of silo milk contamination for each scenario of paratuberculosis prevalence. Dilution significantly reduced the concentration of MAP. The average concentrations of MAP were 0.08 (0.04 SD), 0.14 (0.06 SD), and 0.18 (0.07 SD) log CFU per litre of milk in the silo.

The pasteurisation process also reduced the milk’s MAP contamination by 4 to 7 log CFU. The result of pasteurisation and modelling MAP concentration per litre of milk was that not a single litre of pasteurised milk at the industry level (*MAP_industry past_*) was found to contain MAP cells. 

It is believed that UHT treatment of milk completely deactivates MAP [47], resulting in zero MAP CFU per litre of UHT milk. Therefore, contamination of UHT milk was not included in the simulation model.

### 3.3. Risk of Exposure to MAP via Milk for the Average Consumer

In Slovenia in 2018, the average annual consumption of all types of milk combined was 43 litres per household member. 

The risk of exposure to MAP is highest when consuming raw milk. For example, if the consumer buys milk from a MAP positive farm or the farmer and their family drink the same raw milk, there is an 86.67% chance that the litre of milk they consume contains MAP cells and less than a 1.82% chance that there are more than 100 MAP CFU per litre of milk. More specifically, there is a 100% chance of being exposed to viable MAP in a year. However, if the milk is pasteurised at the MAP positive farm, there is only a 2.97% chance that they will be exposed to MAP in a year via the milk, and none of the contaminated litres of milk would contain more than 2 MAP cells. 

At the dairy industry level, in all three herd-level prevalence scenarios, our model showed that the probability of exposure to MAP through pasteurised milk was zero. Even if the milk was contaminated in a silo, the dilution process reduced MAP concentration enough for the pasteurisation process to mitigate the contamination. 

Based on data on on-farm consumption of milk and direct sales of raw milk, as well as market shares of pasteurised and UHT retail milk, and assuming that the average consumer consumes all these types of milk, we calculated that the average Slovenian consumer consumes 3.24 litres of raw milk, 3.94 litres of pasteurised retail milk, and 35.82 litres of UHT milk. Since the probability of exposure to MAP via raw milk from positive farms was high, taking into account the proportions of different types of milk on the market and the random distribution of positive herds, the probability of exposure to a small amount of viable MAP via milk in Slovenia was 21–49%.

### 3.4. Sensitivity Analysis

**Figure 5:**
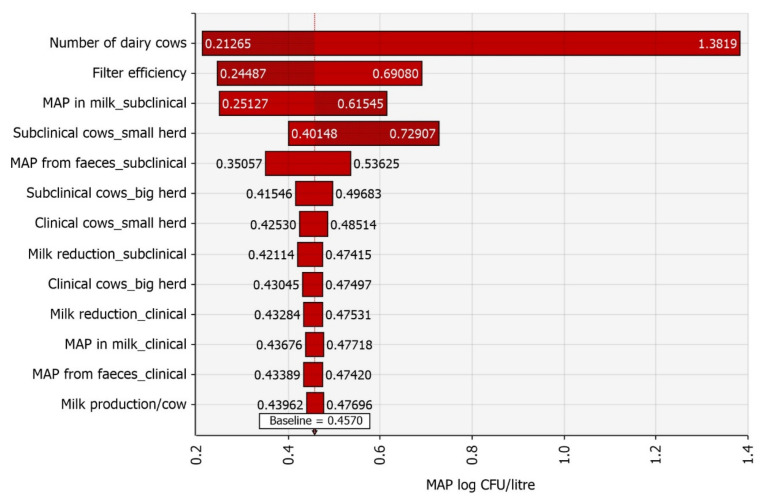
Input variables ranked by the effect on the mean raw bulk tank milk contamination with *Mycobacterium avium* subsp. *paratuberculosis* (MAP).

The farm-level sensitivity analysis results are presented in a tornado diagram (Figure 5). Regardless of which method was used to rank the input variables, whether based on the change in the mean value of raw bulk tank milk contamination, regression coefficients, correlation coefficients, or contribution to variance, the top five input variables at the farm level were always the same. The inputs with the most significant influence on raw milk contamination were the number of dairy cows per herd (Spearman’s ρ = −0.55), filter efficiency (Spearman’s ρ = −0.35), MAP in milk from subclinical cows (Spearman’s ρ = 0.30), number of subclinical cows in a small herd (Spearman’s ρ = 0.26), and MAP from faeces per subclinical cow (Spearman’s ρ = 0.14). All other input variables contributed 0.1% or less to the variance in raw bulk tank milk contamination with MAP. 

**Figure 6:**
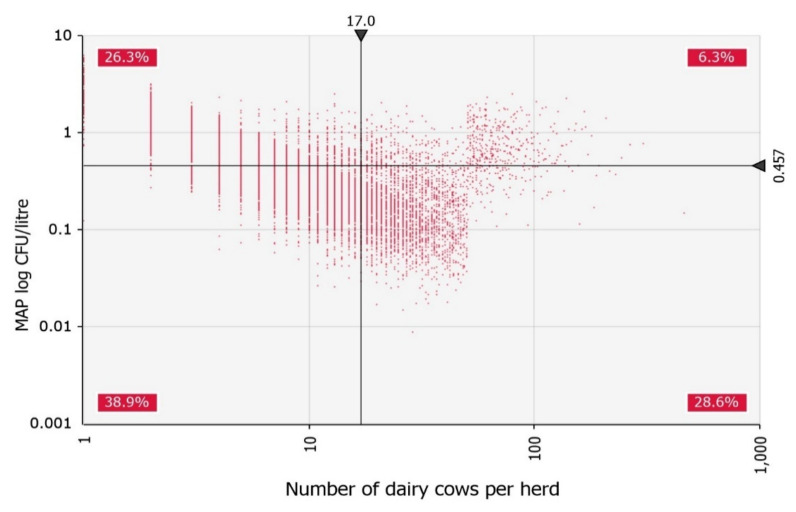
Correlation between *Mycobacterium avium* subsp. *paratuberculosis* (MAP) contamination of raw bulk tank milk and the number of dairy cows per MAP positive herd.

The detailed effect of the number of dairy cows per herd on raw milk contamination with MAP is shown in Figure 6, where the threshold for the small herd (50 cows) can be noticed. It can be seen from the graph that moving the threshold downwards leads to a lower slope of the regression line and consequently to a correlation that is still negative but closer to zero. Conversely, moving the limit upwards leads to a more negative coefficient.

**Figure 7:**
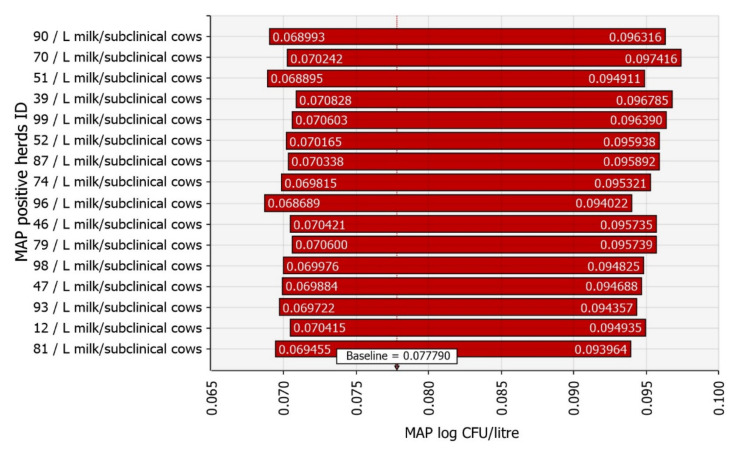
Input variables ranked by the effect on the mean *Mycobacterium avium* subsp. *paratuberculosis* (MAP) contamination of silo milk in scenario 1. The numbers on the *y*-axis indicate the sequential number of simulated dairy herds. In addition, we tested the influence of small herd size threshold values on the model results. We tested four different threshold values based on the proportion of small herds. Specifically, 79.8% of all herds in Slovenia have a maximum of 25 cows, 90% have a maximum of 36 cows, 98.4% have a maximum of 75 cows and 99.3% of all herds have a maximum of 100 cows in a dairy herd. The results are shown in Table 3 with the mean, 5th and 95th PC for milk contamination and human exposure with the proportion of MAP positive litres of milk. In general, lower threshold values resulted in higher contamination of milk with MAP and slightly higher human exposure at the farm level. However, they did not affect the risk of human exposure to MAP via retail milk.

In all three industry level scenarios, the milk produced by subclinical cows on MAP positive farms contributed the most to milk contamination. The example tornado diagram is shown for scenario 1 in Figure 7. 

### 3.5. Risk Characterisation

The summary of our results can be found in Table 4. In a MAP positive dairy herd, MAP concentration per litre of raw bulk tank milk was relatively low. Still, MAP was present in a large proportion of the milk produced, making human exposure almost certain. The amount of MAP in the milk was substantially reduced by the pasteurisation process, making exposure unlikely, which means it could still occur at some point. We estimated that the risk of exposure to MAP is high for those who consume raw milk and dairy products from farms with paratuberculosis. 

On the other hand, the risk of exposure to MAP via milk collected and processed by the dairy industry is most likely negligible. The dilution of MAP concentration in the milk silo, where milk from different dairy farms is mixed together, was sufficient to inactivate MAP cells by pasteurisation. Since a couple of MAP cells could still be found, we indicated the likelihood of exposure as rare. This means that it may occur under exceptional circumstances. 

Based on the consumption and market share of the different types of milk, we determined that the probability of exposure to MAP is possible for the average Slovenian consumer in a year, i.e., it could or should occur at a given time. The amount of MAP that the average consumer could be exposed to is small. Therefore, we estimated the overall risk of exposure to be low. 

## 4. Discussion

This study aimed to assess the risk of exposure to MAP via different types of milk for the Slovenian consumer. The main reason for this is the increasing public health concern and the ongoing efforts to assess and subsequently reduce human exposure to pathogens via food of animal origin. The second reason is the important contribution of the dairy sector to the Slovenian agricultural industry [50]. It is well known that paratuberculosis causes significant direct disease losses in the cattle industry [14] and consequently puts pressure on one of the most important parts of Slovenian agriculture. In addition, the cattle industry may be exposed to indirect disease losses through possible trade bans. In 2018, Slovenia exported milk and dairy products worth more than 169 million euros and imported them worth more than 173 million euros. We assumed that the prevalence of paratuberculosis in Slovenia is now higher than estimates from a decade ago. Therefore, we wanted to estimate the potential risk for human exposure despite the lack of empirical data.

Numerous studies have been published on empirical estimation or modelling of the presence of MAP in milk and dairy products [43,58]. However, no such estimates have been attempted in Slovenia. Because our compartmental model [52] suggested that there might be an essential difference between modelling small and large herds, we developed a stochastic quantitative risk assessment model using Monte Carlo simulations to estimate the extent of milk contamination and the amount of milk produced in MAP infected Slovenian dairy herds.

For input variables based on Slovenian data and the results from our two previous models [52,53], we selected distributions using the AIC selection criterion. Simpler probability distributions such as triangular and uniform distributions were used for other input variables based on the literature and expert opinions. Exceptions were the probability distributions for indirect contamination of milk with MAP from faeces and MAP reduction by pasteurisation, where we used unmodified distributions from previous studies. For indirect contamination of milk with MAP from faeces, separately for subclinically and clinically infected cows, we used the distributions used by Beaunée et al. [59]. Another way to model indirect MAP contamination of milk was to model MAP excretion in faeces, contamination of milk with faeces or dirt, and calculate indirect contamination of milk with MAP from faeces or dirt. According to the expert, it was better to simplify the model because no exact data were available, and we did not have estimates for these steps from Slovenian data. We used the same distribution as Serraino et al. [62] for the MAP reduction with pasteurisation, as we thought their reasoning was grounded. They explained that they took a conservative approach because viable MAP was still found in some experiments despite the reported reduction of more than 8 log.

We modelled the proportion of subclinically and clinically infected animals in a herd in two ways based on the expert opinion. When the model simulated the number of cows corresponding to a small herd, the proportion of infected animals was calculated using the results of our compartmental model; otherwise, the ratio was calculated using data from the literature. This also reflects one of the conclusions of our compartmental model [52], which was that within-herd spread dynamics of MAP depends on herd size. Furthermore, since the results of the compartmental model showed a very weak correlation between the proportion of subclinically and clinically infected cows, we kept these two input variables independent in the exposure estimation model.

The model’s threshold for a small herd was set at 50 cows. We tested the influence of the threshold value on the results of the model. We found that lower thresholds resulted in slightly higher human exposure at the farm level but did not affect the risk of human exposure to MAP via retail milk in any of the prevalence scenarios, because mixing milk from different farms in the milk silo significantly reduced the MAP concentration, so the pasteurisation process mitigated the contamination. When lower thresholds were applied, the higher farm-level exposure resulted from using a probability distribution of MAP prevalence for larger herds in a higher proportion of herds, which assumes a higher MAP prevalence than the probability distribution of MAP prevalence in small herds (Figure 6). Similarly, the threshold value affected the correlation between the number of dairy cows per herd and the MAP concentration in raw bulk tank milk. A threshold value of 50 cows resulted in a moderate negative correlation, while 25 cows lowered the correlation to weak (Spearman’s ρ = −0.22). The negative correlation was not surprising because we modelled MAP contamination of milk in a positive herd, meaning that the minimum number of infected cows is one and in small herds, one animal represents a higher proportion of the herd. When considering the number of infected animals per herd, the relationship between prevalence and MAP contamination of raw bulk tank milk was monotonic and positive for all threshold values tested.

In the sensitivity analysis, besides the number of cows per herd, the other important input variables were milk filter efficiency and variables related to subclinically infected cows. We assumed a uniform distribution from zero to the value used by Rani et al. [58] for milk filter efficiency, based on Van Kessel et al. [61]. They studied the faecal prevalence of Salmonella. The reason for modelling this range of values was that MAP is small and can pass through a filter. Nevertheless, it tends to form clumps that a filter could remove, but we had no data on Slovenia’s situation. Subclinically infected cows and their internal and external milk contamination are more important than variables related to clinically infected cows, as variables related to clinically infected cows contribute little to overall milk contamination with MAP (Figure 5). Subclinically infected cows are more important because they are more common and produce more milk. Thus, if one wanted to reduce the amount of MAP in raw bulk tank milk, culling clinically infected cows would not be sufficient. 

The main pitfalls of the risk assessment carried out are the assumption of a closed market since we have excluded the import and export of milk and dairy products, the random selection of farms supplying milk to a dairy, and the existence of an average consumer.

In 2018, Slovenia was a net exporter of milk and sour milk, while it was a net importer of other dairy products. The total production of drinking milk was 168,000 tons. Almost 307,000 tons of milk were exported in the same year, and just over 44,000 tons were imported. In 2018, nearly 44,000 tons of soured milk were produced, of which 16,000 tons were exported, and 13,000 tons were imported. Only about 16,500 tons of cheese were produced. More than 10,000 tons of cheese were exported, while nearly 27,000 tons were imported. Production of other dairy products was much lower and import mostly exceeded export [55]. These figures suggest that for a more accurate assessment of the risk of exposure to MAP via milk and dairy products for the Slovenian consumer, the risk for each importing country should also be investigated. However, in doing so, one would face similar uncertainties and lack of data and would have to make certain assumptions as we did in our study.

The assumption of a random selection of farms supplying milk to a dairy was made because we had no data on which farms supply milk to which dairy. In the real world, the delivery of milk is not random. Thus, some clusters of MAP positive farms that supply milk to the same dairy might form, either because of trade or shared pastures. Depending on the prevalence at the herd level and the size of the milk silo at the dairy, it could be that the contamination of the milk in the silo is higher than we estimated but the opposite could also be true.

We have assumed an average consumer since more detailed data were not available. The advantage of this assumption is the possibility of estimating the overall exposure of the Slovenian consumer but the results have limited practical value. This is why we also showed the results and reported the risk level separately for each type of milk we were able to evaluate. Nevertheless, MAP is assumed to pose a higher health risk for specific subgroups of the population, so an assessment of the exposure risk for these subgroups would be of interest.

The main uncertainties associated with the risk assessment data are the epidemiological characteristics of MAP. Specifically, the uncertainties are as follows: prevalence of paratuberculosis within the herd and prevalence at the herd level, routes of transmission for infections of animals and humans, infectious dose for humans or different doses for different subgroups if MAP is assumed to be a zoonosis, diagnostic methods to detect MAP, and importance of exposure through the milk and dairy products compared to other possible routes of infection. We overcome these uncertainties to some extent with data from the published literature, some of which are based on empirical studies, while others are based on various models and their calibration. Because of the uncertainties, we narrowed the scope of our research to assess the risk of exposure rather than the risk to human health. Although there are many studies on the presence of MAP in different types of milk and dairy products, we could quantitatively estimate the risk only for raw milk, pasteurised whole milk, and UHT milk. There were not enough data available to quantitatively assess the likelihood and quantity of MAP in other types of milk and dairy products. Nevertheless, we believe that the results are partially generalisable to other dairy products at the appropriate production level.

The main uncertainty related to the assessment methodology is the calibration and validation of the models with independent data, as these are not yet available. Nevertheless, this was the main reason why we chose the presented approach. The models were checked through an internal validation and sensitivity analysis, and the assumptions on MAP, the choice of parameters used, and the feasibility of the results obtained were reviewed by the experts. 

At the farm level, two possible exposures to MAP were assessed in a MAP positive farm, namely possible exposure from raw milk and pasteurised milk. The results showed a high percentage of raw milk with viable MAP cells. This means that for consumers drinking raw milk and consuming raw milk products from farms with paratuberculosis, the risk of exposure to MAP was high. If the milk was pasteurised on the farm, the risk of exposure was significantly reduced but not completely mitigated. Therefore, for consumers drinking pasteurised milk from the farm and consuming products made from pasteurised milk or further processed products, the risk of exposure to MAP was low. However, if the prevalence of paratuberculosis within the farm were to be higher than assumed in our model, the risk of exposure to MAP via pasteurised milk from the positive farm would also increase.

At the dairy industry level, the risk of exposure to MAP has also been assessed for two types of milk, pasteurised and UHT. After pasteurisation of the milk, MAP was mostly inactivated, but still not completely. However, as the number of MAP cells per litre of milk was very low, we assessed the risk of exposure as insignificant. Our results are in agreement with the conclusions of other studies [45,65] that the required pasteurisation time and temperature regimes are sufficient to inactivate possible MAP cells contaminating the milk in the dairy plant. However, this might not be the case if the prevalence at animal or herd level is higher than assumed in our model, and given the rising prevalence of paratuberculosis, this may already be the case in some countries [41]. Since other dairy products are further processed and MAP does not replicate outside the host, we assumed that exposure to MAP via milk and dairy products in Slovenia was rare at the dairy industry level. Therefore, we assessed that the risk of exposure to MAP via milk collected and processed by the dairy industry is most likely insignificant. 

Considering the underlying assumptions and uncertainties, our risk assessment model has shown that the overall risk of exposure to MAP via milk and dairy products is low for the average Slovenian consumer. Nevertheless, our results should be of concern. This is especially true for people with pre-existing conditions and infants, as buying raw milk from milk vending machines or from on-farm sales and homemade dairy products is quite common in Slovenia. Vulnerable groups are advised to avoid consuming raw milk or boil the raw milk before consumption or to consume pasteurised milk from trusted sources or UHT milk.

## 5. Conclusions

This study has shown that despite the lack of empirical data on MAP epidemiological characteristics, comprehensive insights can be gained. However, this is the major gap that future studies should address. The model relies on several assumptions, yet we believe it sufficiently reflects the Slovenian situation and the current state of knowledge. Considering the trends in consumption of dairy products, the increasing paratuberculosis prevalence, and the growing body of scientific literature linking MAP to human diseases, our model could serve as a decision support tool. It is important for other countries or regions with a similar structure of dairy herds. Since exposure to MAP via different types of milk is only one of the possible routes of exposure, without knowing the importance compared to the others, that the overall risk is not negligible, and that risk reduction via this route seems trivial, this study provides a good starting point for possible risk reduction efforts. In addition to heat treatment of milk, efforts should also be made to reduce the paratuberculosis prevalence in cattle herds. This would not only benefit consumers but would also be a preventive measure for the livelihood and welfare of farmers, as a potential public health scare could be avoided if the zoonotic potential of MAP is confirmed.

## Figures and Tables

**Table 1:** Probability distributions of the input variables used in the model to assess human exposure to *Mycobacterium avium* subsp. *paratuberculosis* (MAP) from different types of milk.

Symbol	Description	Probability Distribution	Source
Herd level prevalence (S_i_)			
$S_{1}$	Herd level prevalence, best-case scenario (%)	$S_{1}=Normal\left(21.2991,\;0.10283\right)$	[53]
$S_{2}$	Herd level prevalence, middle scenario (%)	$S_{2}=Normal\left(38.88523,\;0.31328\right)$	[53]
$S_{3}$	Herd level prevalence, estimated scenario (%)	$S_{3}=Normal\left(49.21046,\;0.32587\right)$	[53]
Within-herd level prevalence			
N	Number of dairy cows per herd	$N=Pearson6\left(2.2799,3.8476,21.026\right)$	[56]
$P_{s}$	Proportion of subclinically infected cows	$P_{s}=\left\{\begin{array}{c} Expon\left(0.064147,\;Truncate\left(0,0.5\right)\right),\;if\;N<50\\ Triang\left(0.051,0.15,0.51\right),\;\;\;\;\;\;\;\;\;\;\;\;\;\;\;\;\;\;\;\;\;otherwise \end{array}\right.$	[52,58]; expert opinion
$P_{c}$	Proportion of clinically infected cows	$P_{c}=\left\{\begin{array}{c} Expon\left(0.0075005,\;Truncate\left(0,0.1875\right)\right),\;if\;N<50\\ Triang\left(0.009,0.018,0.022\right),\;\;\;\;\;\;\;\;\;\;\;\;\;\;\;\;\;\;\;\;\;\;\;\;\;\;otherwise \end{array}\right.$	[52,58]; expert opinion
Milk production			
$m_{h}$	Milk production per healthy cow (litres/day)	$m_{h}=Lognorm(25.503,\;5.1687,\;Shift\left(-4.9786\right),\;$ $Truncate\left(15,\;60\right))$	[56]
$\eta_{s}$	Proportion of milk produced per subclinical cow	$\eta_{s}=Triang\left(0.88,\;0.94,\;0.96\right)$	[58,59]; expert opinion
$\eta_{c}$	Proportion of milk produced per subclinical cow	$\eta_{c}=Triang\left(0.35,\;0.75,\;0.85\right)$	[60]; expert opinion
Milk contamination with MAP			
$MAP_{ms}$	MAP shedding in milk per subclinical cow (log CFU/litre)	$MAP_{ms}=Triang\left(0,\log_{10}5\times 10^{4},\log_{10}8.8\times 10^{4}\right)$	[58,59]; expert opinion
$MAP_{mc}$	MAP shedding in milk per clinical cow (log CFU/litre)	$MAP_{mc}=Triang\left(0,\log_{10}(5\times 10^{4}),\log_{10}\left(8.8\times 10^{4}\right),\;Truncate(1.6,\log_{10}\left(8.8\times 10^{4}\right))\right)$	[58,59]; expert opinion
$MAP_{fs}$	MAP from faeces per subclinical cow (log CFU/litre)	$MAP_{fs}=log_{10}\left(1+10^{3}\cdot Beta\left(1,\;25\right)\right)$	[59]
$MAP_{fc}$	MAP from faeces per clinical cow (log CFU/litre)	$MAP_{fc}=log_{10}\left(10^{3+10\cdot Beta\left(50,\;\;200\right)}\right)$	[59]
φ	Milk filter efficiency	$\varphi =Uniform\left(0,\;0.67\right)$	[58,61]; expert opinion
ε	MAP reduction with pasteurisation (log CFU)	$\varepsilon =Uniform\left(4,\;7\right)$	[62]

**Table 2:** Calculations used in the model to assess human exposure to *Mycobacterium avium* subsp. *paratuberculosis* (MAP) from different types of milk.

Symbol	Description	Calculation
HS	Herd status	$HS=Bernoulli(S_{i})$
$N_{s}$	Number of subclinically infected cows	$N_{s}=\left\{\begin{array}{ll} P_{s}\cdot N, & if\;P_{s}\cdot N+N_{c}>0\\ 1, & otherwise \end{array}\right.$
$N_{c}$	Number of clinically infected cows	$N_{c}=P_{c}\cdot N$
$N_{h}$	Number of healthy cows	$N_{h}=N-N_{s}-N_{c}$
Milk production		
$m_{s}$	Milk per subclinically infected cow (litres/day)	$m_{s}=m_{h}\cdot \eta_{s}$
$m_{c}$	Milk per clinically infected cow (litres/day)	$m_{c}=m_{h}\cdot \eta_{c}$
m	Milk per herd (litres/day)	$m=\overset{N_{h}}{\underset{i=1}{\sum }}m_{h,i}+\overset{N_{s}}{\underset{j=1}{\sum }}m_{s,j}+\overset{N_{c}}{\underset{k=1}{\sum }}m_{c,k}$
Milk contamination with MAP		
$MAP_{int}$	Internal milk contamination (log CFU)	$MAP_{int}=\overset{N_{s}}{\underset{j=1}{\sum }}m_{s,j}\cdot MAP_{ms}+\overset{N_{c}}{\underset{k=1}{\sum }}m_{c,k}\cdot MAP_{mc}$
$MAP_{ext}$	External milk contamination (log CFU)	$MAP_{ext}=\overset{N_{s}}{\underset{j=1}{\sum }}m_{s,j}\cdot MAP_{fs}+\overset{N_{c}}{\underset{k=1}{\sum }}m_{c,k}\cdot MAP_{fc}$
$MAP_{herd}$	Bulk tank raw milk contamination on farm level (log CFU)	$MAP_{herd}=\left(\frac{MAP_{int}+MAP_{ext}}{m}\right)\cdot \left(1-\varphi \right)$
$MAP_{herd\;raw}$	Raw milk contamination on farm level (CFU/litre)	$MAP_{herd\;past\;}=Poisson(10^{MAP_{herd}})$
$MAP_{herd\;past\;}$	Pasteurised whole milk contamination on farm level (CFU/litre)	$MAP_{herd\;past\;}=Poisson(10^{MAP_{herd}-\varepsilon })$
$MAP_{industry}$	Silo milk contamination on dairy industry level (log CFU)	$MAP_{industry}=\sum_{i=1}^{100}MAP_{herd,i}$
$MAP_{industry\;past\;}$	Pasteurised whole milk contamination on dairy industry level (CFU/litre)	$MAP_{industry\;past\;}=Poisson(10^{MAP_{industry}-\varepsilon })$

**Table 3:** Results of sensitivity analysis of small herd size thresholds in the risk assessment model of human exposure to *Mycobacterium avium* subsp. *paratuberculosis* (MAP) via different types of milk.

	the Threshold Value for the Number of Cows in a Small Herd				
	25 Cows	36 Cows	Basic Mode l50 Cows	75 Cows	100 Cows
Milk contamination	Mean (5th, 95th PC)				
$MAP_{herd}$ (log CFU/L)	0.53 (0.1, 1.45)	0.48 (0.08, 1.37)	0.46 (0.07, 1.31)	0.43 (0.07, 1.28)	0.43 (0.07, 1.28)
$MAP_{herd\;raw}$ (CFU/L)	175.28 (0, 28)	166.55 (0, 24)	164.75 (0, 22)	132.12 (0, 20)	136.53 (0, 20)
$MAP_{herd\;past\;}\;$(CFU/L)	0.001 (0, 0)	0.001 (0, 0)	0.001 (0, 0)	0.0008 (0, 0)	0.001 (0, 0)
$MAP_{industry}\;$scenario 1 (log CFU/L)	0.11 (0.04, 0.21)	0.09 (0.03, 0.18)	0.08 (0.03, 0.16)	0.07 (0.03, 0.14)	0.06 (0.03, 0.12)
$MAP_{industry}\;$scenario 2 (log CFU/L)	0.2 (0.1, 0.34)	0.17 (0.08, 0.29)	0.14 (0.07, 0.25)	0.12 (0.06, 0.22)	0.11 (0.06, 0.2)
$MAP_{industry}\;$scenario 3 (log CFU/L)	0.26 (0.14, 0.42)	0.21 (0.11, 0.36)	0.18 (0.09, 0.31)	0.15 (0.08, 0.27)	0.14 (0.08, 0.24)
$MAP_{industry\;past\;}$(CFU/L)	0 (0, 0)	0 (0, 0)	0 (0, 0)	0 (0, 0)	0 (0, 0)
**Human exposure**	**%**				
Raw milk in a positive farm with >0 MAP CFU/L	89.13	87.58	86.67	85.98	85.82
Raw milk in a positive farm with >100 MAP CFU/L	2.05	1.90	1.83	1.82	1.84
Pasteurised milk in a positive farm with >0 MAP CFU/L	0.09	0.09	0.08	0.06	0.06
Pasteurised milk at the industry level with >0 MAP CFU/L	0	0	0	0	0

**Table 4:** Summary of risk characterisation by milk type and overall exposure of the average Slovenian consumer to *Mycobacterium avium* subsp. *paratuberculosis* (MAP).

	Likelihood of Exposure	Level of MAP CFU/L of Milk	Level of Risk	Level of Uncertainty
Raw bulk tank milk in a MAP positive farm	Almost certain	High	High	Low to moderate
Pasteurised milk in a MAP positive farm	Unlikely	Low	Low	Low to moderate
Pasteurised milk at the dairy industry level (all three scenarios)	Rare	Insignificant	Negligible	Low to moderate
UHT milk at the dairy industry level	Insignificant	Insignificant	Negligible	Low
Exposure of the average Slovenian consumer	Possible	Low	Low	Moderate

## Data Availability

The data presented in this study and developed model are available on request from the corresponding author.
